# Prognostic Value of Plasma Epstein-Barr Virus DNA Levels Pre- and Post-Neoadjuvant Chemotherapy in Patients With Nasopharyngeal Carcinoma

**DOI:** 10.3389/fonc.2021.714433

**Published:** 2021-09-16

**Authors:** Lisheng Zhu, Tao Ouyang, Ying Xiong, Li Ba, Qiuting Li, Mengjun Qiu, Zhenwei Zou, Gang Peng

**Affiliations:** ^1^ Cancer Center, Union Hospital, Tongji Medical College, Huazhong University of Science and Technology, Wuhan, China; ^2^ Department of Radiology, Union Hospital, Tongji Medical College, Huazhong University of Science and Technology, Wuhan, China; ^3^ Division of Gastroenterology, Liyuan Hospital, Tongji Medical College, Huazhong University of Science and Technology, Wuhan, China

**Keywords:** nasopharyngeal carcinoma, Epstein-Barr virus DNA, neoadjuvant chemotherapy, prognostic factor, nomogram

## Abstract

**Background:**

In this study, we evaluated the prognostic value of the plasma levels of Epstein-Barr virus (EBV) DNA in patients with nasopharyngeal carcinoma (NPC) at different treatment stages.

**Methods:**

We retrospectively analyzed the Data of 206 patients with NPC. Pre-neoadjuvant chemotherapy (pre-NACT), post-NACT, post-radiotherapy, and post-treatment plasma EBV DNA levels were used to establish prognostic nomograms. The concordance index (C-index) and calibration curves were used to compare the prognostic accuracy of the nomograms. The results were confirmed in a validation cohort consisting of patients who were tested for EBV DNA levels at all four stages of treatment. The Kaplan-Meier method was used to calculate the progression-free survival (PFS) and overall survival (OS). Survival differences were calculated using the log-rank test.

**Results:**

EBV DNA-positive patients had worse 3-year PFS and 5-year OS than EBV DNA-negative patients; this was true for pre-NACT (PFS: 82.7% *vs*. 57.3%, *P* < 0.001; OS: 90.9% *vs*. 68.7%, *P* = 0.08) and post-NACT (PFS: 85.0% *vs*. 50.6%, *P* < 0.001; OS: 91.7% *vs*. 65.7%; *P* = 0.001) EBV DNA levels but not for post-radiotherapy (PFS: 72.2% *vs*. 60.9%, *P* = 0.192; OS: 73.1% *vs*. 77.2%, *P* = 0.472) or post-treatment (PFS: 77.3% *vs*. 59.2%, *P* = 0.063; OS: 77.5% *vs*. 79.7%, *P* = 0.644) levels. Nomograms combining pre-NACT and post-NACT EBV DNA levels had a superior prognostic ability than those of post-radiotherapy and post-treatment EBV DNA levels.

**Conclusion:**

Pre-NACT EBV DNA levels combined with post-NACT EBV DNA levels can more reliably predict survival outcomes in patients with NPC.

## Introduction

Nasopharyngeal carcinoma (NPC) is relatively common in Southeast Asian countries due to the high prevalence of Epstein-Barr virus (EBV) infections (1). The recent advances in intensity-modulated radiotherapy (IMRT) and concurrent chemoradiotherapy (CCRT) have greatly improved the prognosis and survival outcomes of patients with NPC (2). However, some patients with NPC develop local recurrence or metastasis within 2 years of treatment (3). Patients with NPC receive different neoadjuvant chemotherapy (NACT) and adjuvant chemotherapy (ACT) regimens depending on their TNM stage. A phase III clinical trial showed that ACT with cisplatin and fluorouracil did not significantly improve the failure-free survival in patients with a locally advanced NPC (4). However, NACT with gemcitabine and cisplatin increased the 3-year recurrence-free survival from 76.5% to 85.3% in patients with a locally advanced NPC (5). In another study, three cycles of NACT improved the disease-free survival in patients with advanced NPC, although no significant changes were observed in the overall survival (OS) (6). Furthermore, the combination of NACT and ACT had no effect on the distant metastasis-free survival and OS in patients with advanced, high-risk NPC, despite a moderate improvement in the prognosis of low-risk patients (7, 8). Hence, the clinical benefit of NACT and ACT in patients with NPC merits further investigation.

EBV infection is associated with an increased risk of NPC (9–12). The relationship between the EBV DNA levels and the prognosis of patients at different stages of treatment has also been reported (13–16). Pre-treatment and post-treatment EBV DNA levels are considered as an indicator of tumor load and tumor malignancy. Notably, pre-treatment EBV DNA levels in the plasma of patients with NPC were significantly correlated with distant metastasis (6), relapse (17), and long-term OS (18, 19). Additionally, post-radiotherapy EBV DNA levels in the plasma of patients with NPC predicted locoregional failure, distant metastasis, and death (20). Post-treatment plasma EBV DNA levels also predicted distant metastasis (21) and tumor recurrence (22); thus, additional treatment in patients with high post-treatment EBV DNA levels may prevent relapse (14). Many NPC prognostic models are based on the EBV characteristics and serological indicators (19, 23, 24). The predictive ability of the nomograms of the circulating EBV DNA levels is higher than that of nomograms of the TNM stage (25). These findings suggest that plasma EBV DNA levels are useful in risk stratification and prognosis prediction in patients with NPC (23, 26). Furthermore, evaluating the plasma EBV DNA levels may improve the prediction of the PFS and OS (27, 28).

Nevertheless, the relevance of the dynamic changes in EBV DNA levels in NPC prognosis remains unclear. In this study, we evaluated the prognostic value of plasma EBV DNA levels at different treatment stages and the relationship between plasma EBV levels and NACT outcomes. We also developed a nomogram by combining pre-NACT and post-NACT EBV levels with other traditional risk factors.

## Methods

### Patients

**Figure 1:**
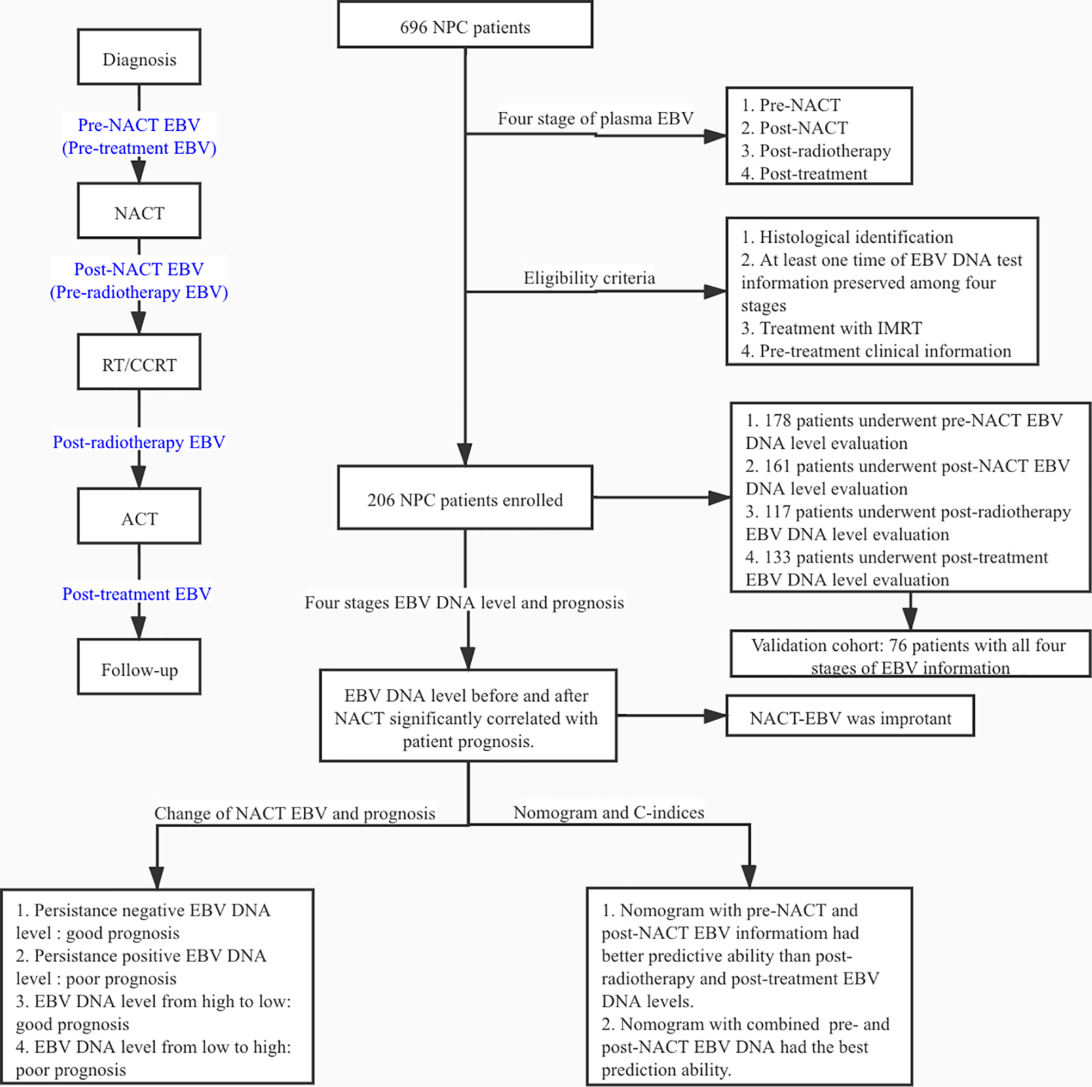
Flow chart of this study.

>We retrospectively reviewed the data of 696 patients diagnosed with NPC who underwent radiotherapy with NACT at Wuhan Union Hospital Cancer Center between July 2012 and October 2018; 490 patients were excluded because of the lack of information on the plasma EBV DNA levels. Among the 206 NPC patients, the plasma EBV DNA levels were evaluated before NACT (hereafter referred to as pre-NACT) in 178 patients, after NACT and before radiotherapy (hereafter referred to as post-NACT) in 161 patients, post-radiotherapy in 118 patients, and post-treatment in 133 patients. There was an intersection between the different groups of patients. The validation cohort consisted of 76 patients with known EBV DNA levels at all four treatment stages. The eligibility criteria were as follows: (1) pathological diagnosis of primary NPC; (2) no history of cancer treatment; (3) at least one plasma EBV DNA level evaluation (pre-NACT, post-NACT, post-radiotherapy, or post-treatment); (4) treatment with IMRT; (5) NACT treatment; (6) availability of baseline clinical data, including routine blood indicators and liver and kidney function. The experimental design is shown in **Figure 1**.

### Diagnosis and Treatment

Baseline demographic and clinicopathological characteristics, including gender, age, and smoking status, were collected for all patients. Blood samples were collected before treatment to assess the levels of white blood cells, hemoglobin (HGB), platelets (PLT), lactate dehydrogenase (LDH), and EBV DNA. The clinical tumor stage was determined according to the AJCC TNM staging guidelines, seventh edition. All patients received IMRT and NACT, with or without adjuvant chemotherapy (ACT). NACT regimens were as follows: 1) 75 mg/m^2^ of docetaxel and 75 mg/m^2^ of cisplatin on day 1, and 750 mg/m^2^ of fluorouracil for 5 days; 2) 1,000 mg/m^2^ of gemcitabine on day 1 and day 8, 80 mg/m^2^of cisplatin on day 1; 3) 75 mg/m^2^ of docetaxel and 75 mg/m^2^ of cisplatin on day 1. Each regimen was given in three-week cycles for a total of three cycles. Details on NACT regimens can be found in **Tables 1** and **S2**.

**Table 1:** Patient demographic and clinical characteristics.

Characteristic	Patients
	No. (%)
**Age, y**	
≤45	91 (44.2)
>45	115 (55.8)
**Gender**	
Female	49 (23.8)
Male	157 (76.2)
**Clinical stage**	
I/II	29 (14.1)
III	87 (42.2)
IVa	74 (35.9)
IVb	16 (7.8)
**Tumor stage**	
T1	3 (1.5)
T2	70 (34.0)
T3	78 (37.9)
T4	55 (26.7)
**Node stage**	
N0	9 (4.4)
N1	57 (27.7)
N2	102 (49.5)
N3	38 (18.4)
**M stage**	
M0	190 (92.2)
M1	16 (7.8)
**Treatment**	
NACT+RT ± ACT	140 (68.0)
NACT+CCRT ± ACT	66 (32.0)
**NACT**	
DCF	112 (54.4)
DP	59 (28.6)
GP	35 (17.0)
**LDH, g/L**	
<245	187 (93.2)
≥245	19 (6.8)
**WBC, 10** ^9^/L	
<4	21 (10.2)
4–10	173 (84.0)
≥10	12 (5.8)
**HGB, g/L**	
<120	33 (16.0)
120–150	133 (64.6)
≥150	40 (19.4)
**PLT, 10** ^9^/L	
<100	3 (1.5)
100–300	174 (84.5)
≥300	29 (14.1)
**Smoking**	
No	111 (53.9)
Yes	95 (46.1)
**Alcohol**	
No	138 (67.0)
Yes	68 (33.0)
**EBV levels**	
Negative	–
Positive	–
**Clinical Outcome**	
No progress	152 (73.8)
Local recurrence	32 (15.5)
Distant metastasis	22 (10.7)
Death	31 (15.0)

NACT, neoadjuvant chemotherapy; RT, radiotherapy; CCRT, concurrent chemoradiotherapy; ACT, adjuvant chemotherapy; DCF, docetaxel plus cisplatin and fluorouracil; DP, docetaxel plus cisplatin; GP, gemcitabine plus cisplatin; LDH, lactate dehydrogenase; WBC, white blood cells; HGB, hemoglobin; PLT, platelets; Positive EBV level was defined as serum EBV DNA load higher than 400 copies/mL; otherwise, was negative. N = 206.

### Plasma Epstein-Barr Virus DNA Evaluation

Plasma EBV DNA levels were assessed by quantitative PCR (qPCR) as previously described (23). Samples with EBV DNA levels higher than 400 copies/mL were considered EBV-positive. As not all patients had been evaluated for the EBV DNA levels at all four stages, we separately assessed patients with available data on pre-NACT, post-NACT, post-radiotherapy, and post-therapy plasma EBV DNA levels.

### Patient Follow-Up

Patients were followed up every three months in the first three years after treatment and every six months thereafter. The primary endpoint of the study was PFS, defined as the time from diagnosis to disease progression or any-cause death. The secondary endpoint was OS, defined as the time from diagnosis to any-cause death. Patients were censored at the last follow-up date (January 2020).

### Statistical Analysis

Statistical analyses were conducted using R version 3.6.3 (http://www.R-project.org). The Kaplan-Meier method was used to calculate the PFS and OS; survival differences were compared using the log-rank test. Patient characteristics were compared using the χ^2^ or Fisher’s exact test. Significant factors in univariate analysis were used in the multivariable Cox regression analysis. A prognostic nomogram was established, and the concordance index (C-index) and calibration curve were used to determine the accuracy and discriminative ability of the nomogram. Two-sided *P*-values < 0.05 were considered statistically significant.

## Results

### Patient Characteristics

In this retrospective study, we analyzed the data of 206 patients with NPC. There were no differences in the baseline characteristics between the included and excluded patients (**Table S1**). Plasma EBV DNA levels were determined pre-NACT in 178 patients, post-NACT in 161 patients, post-radiotherapy in 118 patients, and post-treatment in 133 patients. The patient demographics and clinical characteristics are presented in **Table 1**. The median follow-up time was 36.4 months (range, 3.8–91.6 months). A total of 54 (26.1%) patients had recurrent disease and distant metastasis, and 31 (15.0%) patients died. All patients received NACT before radiotherapy or CCRT.

### Plasma Epstein-Barr DNA Levels Before and After Neoadjuvant Chemotherapy Are Significantly Associated With Patient Prognosis

**Figure 2:**
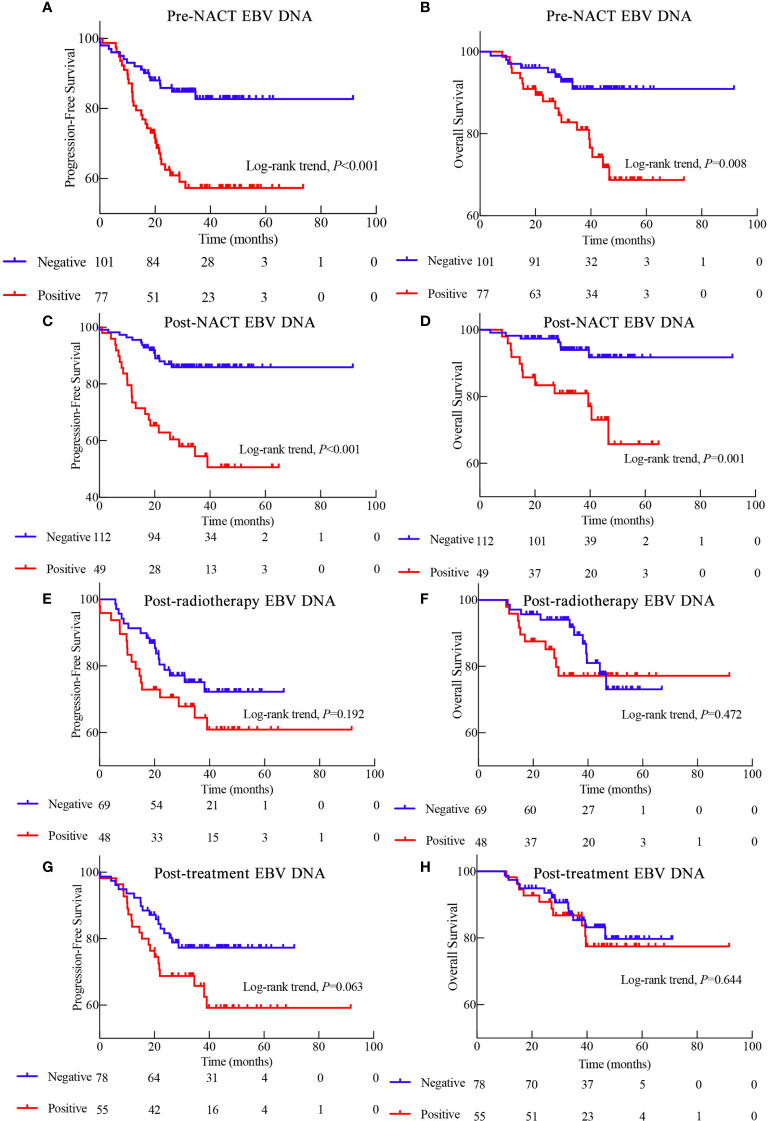
Kaplan-Meier survival curves showing the progression-free survival (PFS) and overall survival (OS) of patients with EBV tested during different stages of treatment. **(A)** PFS based on pre- neoadjuvant chemotherapy (NACT) EBV DNA levels. **(B)** OS based on pre-NACT EBV DNA levels. **(C)** PFS based on post-NACT EBV DNA levels. **(D)** OS based on post-NACT EBV DNA levels. **(E)** PFS based on post-radiotherapy EBV DNA levels. **(F)** OS based on post-radiotherapy EBV DNA levels. **(G)** PFS based on post-treatment EBV DNA levels. **(H)** OS based on post-treatment EBV DNA levels.

The 3-year PFS rate of EBV DNA-negative patients before NACT was significantly higher than that of EBV DNA-positive patients (82.7% *vs*. 57.3%, *P* < 0.001; **Figure 2**). The risk of disease progression in patients positive for EBV DNA after NACT was 4.105 times (95% CI, 1.975–8.533) higher than that of patients negative for EBV DNA after NACT. Additionally, the 3-year PFS and 5-year OS rates were significantly lower in patients positive for EBV DNA after NACT than in EBV DNA-negative patients (3-year PFS: 50.6% *vs*. 85.0%, *P* < 0.001; 5-year OS: 91.7% *vs*. 65.7%; *P* = 0.001). There were no significant differences in the 3-year PFS and 5-year OS between patients stratified by post-radiotherapy EBV DNA levels (3-year PFS: 72.2% *vs*. 60.9%, *P* = 0.192; 5-year OS: 73.1% *vs*. 77.2%, *P* = 0.472). The prognosis of patients with high post-treatment EBV DNA levels tended to be worse than that of patients with low EBV DNA levels (3-year PFS: 59.2% *vs*. 77.3%, *P* = 0.063; 5-year OS: 77.5% *vs*. 79.7%, *P* = 0.644).

**Figure 3:**
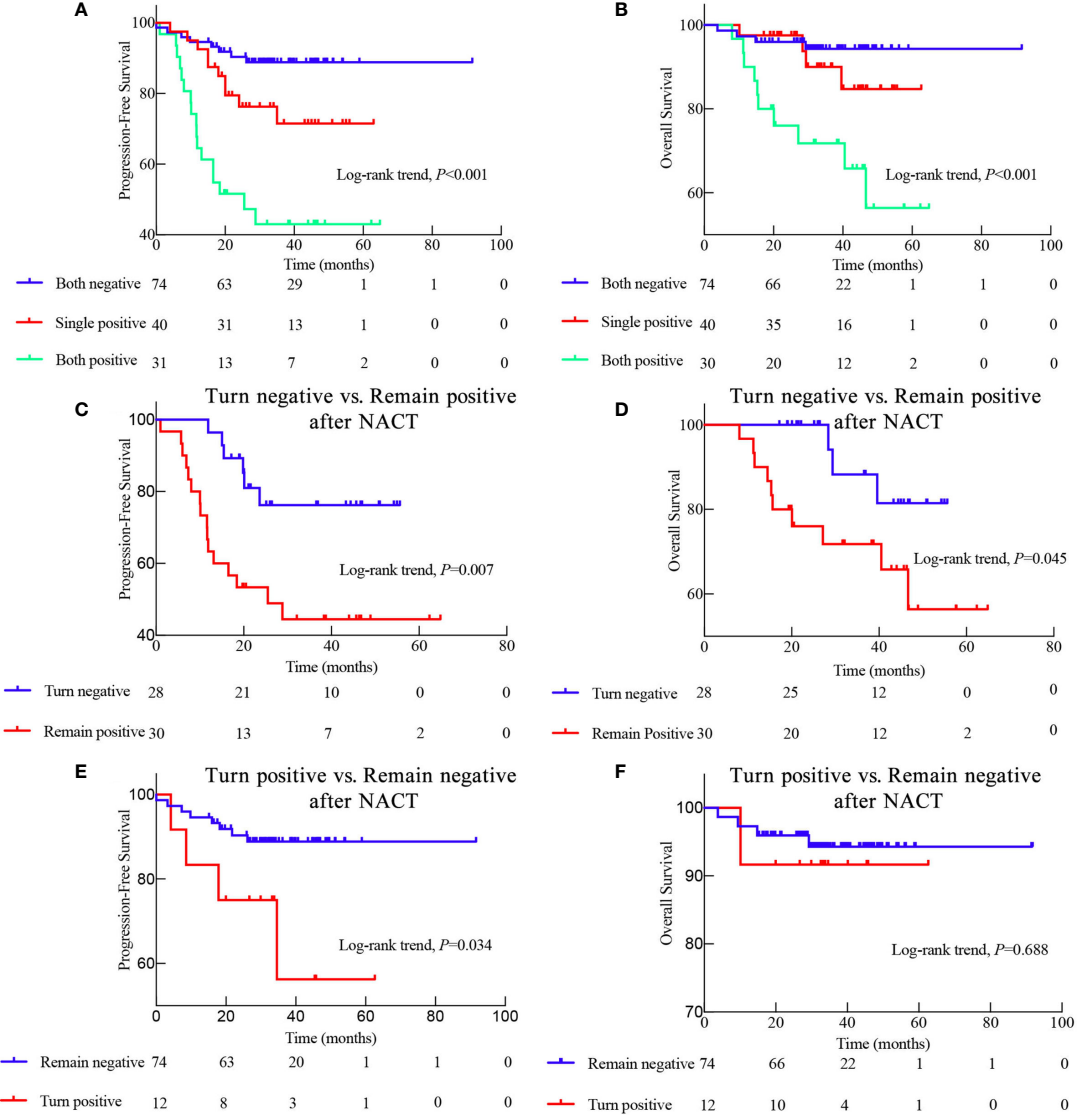
Kaplan-Meier survival curves displaying the progression-free survival (PFS) and overall survival (OS) of patients with different EBV status during neoadjuvant chemotherapy (NACT). **(A)** PFS and **(B)** OS of patients with different EBV status throughout NACT as follows: Both negative means EBV DNA-negative before and after NACT; Single positive means EBV DNA-positive either before or after NACT; Both positive means EBV DNA-positive both before and after NACT; **(C)** Comparison of the PFS and **(D)** OS between patients whose EBV DNA status switched from positive to negative after NACT and those who remained EBV DNA-positive after NACT; **(E)** Comparison of the PFS and **(F)** OS between patients with EBV DNA status changing from negative to positive and those who remained EBV DNA-negative after NACT.

Because pre-NACT and post-NACT EBV DNA levels were strongly associated with PFS, we further investigated the relationship between NACT-associated EBV DNA levels and patient prognosis. Interestingly, the prognosis of patients with an EBV DNA-negative status all along was significantly better than that of patients who were EBV DNA-positive either before or after NACT and who were EBV DNA-positive both before and after NACT (3-year PFS: 88.8% *vs*. 71.5% *vs*. 40.1%, P < 0.001; 5-year OS: 94.3% *vs*. 84.7% *vs*. 56.4%, P < 0.001; **Figures 3A, B**).

### Switch From Epstein-Barr Virus DNA-Positive Level to Negative Epstein-Barr Virus DNA Level After Neoadjuvant Chemotherapy Is Associated With a Favorable Prognosis

We also evaluated the prognostic significance in the conversion from EBV DNA-positive to EBV DNA-negative after NACT. The prognosis of patients who exhibited a conversion from EBV DNA-positive to EBV DNA-negative after NACT was significantly better than that of patients who remained EBV DNA-positive (3-year PFS: 76.2% *vs*. 44.4%, *P* = 0.007; 5-year OS: 81.4% *vs*. 56.4%, *P* = 0.045; **Figures 3C, D**). Consistently, the 3-year PFS was significantly worse in patients who displayed conversion from EBV DNA-negative to EBV DNA-positive after NACT than in those who remained EBV DNA-negative (3-year-PFS: 56.3% *vs*. 88.8%, *P* = 0.034; **Figures 3E, F**). However, the prognostic ability of the conversion from EBV DNA-negative to EBV DNA-positive, and vice versa, after RT and ACT, was less profound (**Figure S1**). These findings suggest that the changes in the EBV DNA levels during NACT may be a better predictor of prognosis than the changes in the EBV DNA levels during RT or ACT.

We also evaluated whether the plasma EBV DNA levels were affected by the different NACT regimens. We observed no significant differences in the plasma EBV DNA levels based on the NACT regimen (**Table 1**). Similarly, there was also no significant difference in the NACT regimens between patients with different EBV levels (**Table S2**). Moreover, the OS and PFS were similar between patients receiving different NACT regimens (**Figure S2**).

### Prognostic Factors in Nasopharyngeal Cancer

Univariate analysis indicated that the TNM stage, LDH levels, HGB levels, pre-NACT EBV DNA levels, and post-NACT EBV DNA levels were associated with the PFS in patients with NPC (**Table 2**). Multivariable analysis using these factors revealed that the TNM stage, LDH levels, HGB levels, pre-NACT EBV DNA levels, and post-NACT EBV DNA levels were independent risk factors predicting treatment failure (**Table 3**).

**Table 2:** Univariate analysis for the progression-free survival of the primary cohort (N = 206).

Variable	Patients	
	HR	*P*
**Gender**		
Female	Reference	
Male	1.409 (0.709–2.800)	0.328
**Age, y**		
≤45	Reference	
>45	1.005 (0.983–1.028)	0.639
**TNM stage**		
I/II	Reference	
III	2.220 (0.501–9.841)	0.294
IVa	7.289 (1.743–30.481)	0.007^**^
IVb	10.361 (2.198–48.834)	0.003^**^
**WBC, 10** ^9^/L		
<4	Reference	
4–10	0.939 (0.400–2.206)	0.886
≥10	1.302 (0.367–4.614)	0.683
**Neutrophil, 10** ^9^/L		
<2	Reference	
2–7	0.969 (0.432–2.176)	0.939
≥7	0.643 (0.245–1.690)	0.370
**Lymphocyte**		
<1	Reference	
1–2	0.803 (0.318–2.026)	0.643
≥2	1.127 (0.326–3.893)	0.850
**PLT, 10** ^9^/L		
<100	Reference	
100–300	~	0.996
≥300	~	0.996
**LDH, U/L**	1.006 (1.003–1.009)	<0.001^***^
**HGB, g/L**		
~119/120–149/150~	0.533 (0.337–0.841)	0.007^**^
**Smoking**		
No	Reference	
Yes	1.008 (0.990–1.026)	0.413
**Alcohol**		
No	Reference	
Yes	1.097 (0.628–1.918)	0.744
**EBV DNA**		
Pre-NACT		
Negative	Reference	
Positive	3.065 (1.646–5.705)	<0.001^***^
Post- NACT		
Negative	Reference	
Positive	3.941 (2.029–7.654)	<0.001^***^
Post-radiotherapy		
Negative	Reference	
Positive	1.608 (0.812–3.184)	0.173
Post-treatment		
Negative	Reference	
Positive	1.869 (0.971–3.598)	0.061

NACT, neoadjuvant chemotherapy. **P < 0.01; ***P < 0.001; bold means statistically significant.

**Table 3:** Multivariate analysis for the progression-free survival of the primary cohort (N = 206).

Variable	Patients	
	HR	*P*
**TNM stage**		
I/II	Reference	
III	1.951 (0.439–8.670)	0.379
IVa	5.929 (1.409–24.946)	0.015^*^
IVb	7.519 (1.575–35.905)	0.011^*^
**LDH, U/L**	1.005 (1.002–1.008)	<0.001^***^
**HGB, g/L**		
~119/120–149/150~	0.546 (0.340–0.878)	0.012^*^
EBV DNA		
Pre-NACT		
Negative	Reference	
Positive	2.461 (1.303–4.645)	0.005^**^
Post- NACT		
Negative	Reference	
Positive	3.783 (1.893–7.561)	<0.001^***^
Post-radiotherapy		
Negative	Reference	
Positive	1.265 (0.617–2.592)	0.521
Post-treatment		
Negative	Reference	
Positive	1.834 (0.927–3.626)	0.081

NACT, neoadjuvant chemotherapy. *P < 0.05; **P < 0.01; ***P < 0.001; bold means statistically significant.

### Prognostic Value of the Nomograms of Pre- NACT and Post-NACT Epstein-Barr Virus DNA Levels

**Figure 4:**
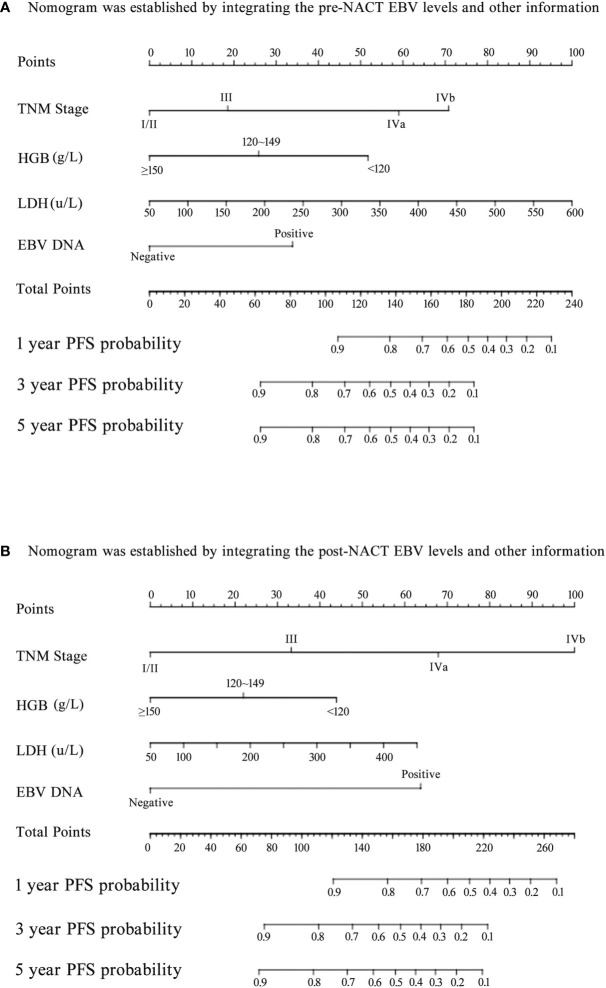
Nomogram for predicting the 1-year, 3-year, and 5-year progression-free survival (PFS) of patients. **(A)** The nomogram was established by integrating the TNM stage, HGB, LDH, and pre-neoadjuvant chemotherapy (NACT) EBV levels; **(B)** The nomogram was established by integrating the TNM stage, HGB, LDH, and post-NACT EBV levels.

**Figure 5:**
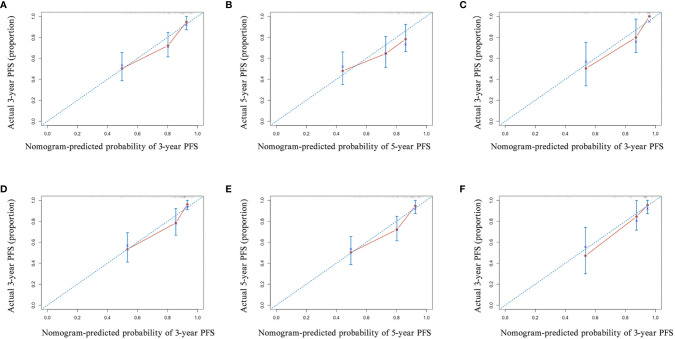
The calibration curve of the nomogram for predicting the progression-free survival (PFS) with pre-neoadjuvant chemotherapy (NACT) or post-NACT EBV DNA levels. **(A)** 3-year and **(B)** 5-year in nomogram with pre-NACT EBV DNA levels in the primary cohort and **(C)** 3-year in the validation cohort; **(D)** 3-year and **(E)** 5-year in nomogram with post-NACT EBV DNA levels in the primary cohort and **(F)** 3-year in the validation cohort. Actual PFS is plotted on the y-axis; nomogram-predicted probability of PFS is plotted on the x-axis.

Next, we established nomograms to predict the PFS in patients with NPC (**Figures 4A, B**; **Figures S3A, B**). Calibration graphs were generated to confirm the accuracy of the prediction model (**Figures 5A, B, D, E**). In these graphs, the x-axes indicated the 3-year or 5-year PFS, and the y-axes indicated the actual survival. The prediction power of the nomograms of pre-NACT and post-NACT EBV DNA levels was higher than that of the nomograms with post-radiotherapy and post-treatment EBV DNA levels, with C-indices of 0.758, 0.780, 0.739, and 0.737, respectively (**Table 4**). The C-indexes of nomogram A and nomogram B were significantly higher than those of EBV DNA levels alone and the TNM staging system, with values of 0.626 (95% CI, 0.555–0.697) and 0.745 (95% CI, 0.697–0.819) in the pre-NACT group and 0.666 (95% CI, 0.588–0.744) and 0.724 (95% CI, 0.655–0.815) in the post-NACT group. We also found that the C-indexes of the nomograms of EBV DNA levels before and after NACT were higher than those of the nomograms of EBV DNA levels after radiotherapy and after treatment. These findings suggest that the plasma EBV DNA levels pre-NACT and post-NACT are promising prognostic factors in patients with NPC.

**Table 4:** The C-indices of nomograms, TNM stage + HGB + LDH, and EBV DNA for the prediction of the progression-free survival (PFS) in the primary cohort and validation cohort .

Factor	Primary cohort		Validation cohort	
	C-index (CI)	*P*	C-index (CI)	*P*
**Pre-NACT**				
Nomogram A	0.758 (0.697–0.819)	<0.001^***^	0.796 (0.704–0.888)	<0.001^***^
Stage+HGB+LDH	0.745 (0.676–0.814)	<0.001^***^	0.736 (0.624–0.848)	0.02^*^
EBV	0.626 (0.555–0.697)	<0.001^***^	0.677 (0.565–0.789)	0.005^**^
**Post-NACT**				
Nomogram B	0.780 (0.713–0.847)	<0.001^***^	0.794 (0.698–0.890)	<0.001^***^
Stage+HGB+LDH	0.724 (0.644–0.804)	<0.001^***^	0.736 (0.624–0.848)	<0.02^*^
EBV	0.666 (0.588–0.744)	<0.001^***^	0.685 (0.573–0.797)	<0.001^***^
**Post-radiotherapy**				
Nomogram C	0.739 (0.657–0.821)	<0.001^***^	0.743 (0.631–0.855)	0.03^*^
Stage+HGB+LDH	0.735 (0.655–0.815)	<0.001^***^	0.736 (0.624–0.848)	0.02^*^
EBV	0.570 (0.484–0.656)	0.1	0.569 (0.447–0.691)	0.2
**Post-treatment**				
Nomogram D	0.737 (0.661–0.813)	<0.001^***^	0.759 (0.663–0.855)	0.03^*^
Stage+HGB+LDH	0.703 (0.621–0.785)	<0.001^***^	0.736 (0.624–0.848)	0.02^*^
EBV	0.579 (0.499–0.659)	0.04^*^	0.543 (0.423–0.663)	0.4

Nomogram A, including four high risk factors (Stage, HGB, LDH, and pre-NACT EBV levels); Nomogram B, including four high risk factors (Stage, HGB, LDH, and post-NACT EBV levels); Nomogram C, including four high risk factors (Stage, HGB, LDH, and post-radiotherapy EBV levels); Nomogram D, including four high risk factors (Stage, HGB, LDH, and post-treatment EBV levels); NACT, neoadjuvant chemotherapy; C-index, concordance index; CI, confidence interval. *P < 0.05; **P < 0.01; ***P < 0.001; bold means statistically significant.

To confirm the prognostic accuracy of the nomogram, we evaluated its prognostic performance in a validation cohort of 76 patients with available data on the EBV load for all four treatment stages. In this validation cohort, the C-indexes of nomograms A and B were higher than those of nomograms C and D (A: 0.796, 95% CI, 0.704–0.888; B: 0.794, 95% CI, 0.698–0.890; C: 0.743, 95% CI, 0.631–0.855; D: 0.759, 95% CI, 0.663–0.855; **Table 4**; **Figures 5C, F** and **Figures S4C, F**). The prognostic power of pre-NACT and post-NACT EBV DNA levels was higher than that of post-radiotherapy and post-treatment EBV DNA levels.

### The Combination of Pre-NACT and Post-NACT Epstein-Barr Virus DNA Levels Improves the Prognostic Accuracy

**Figure 6:**
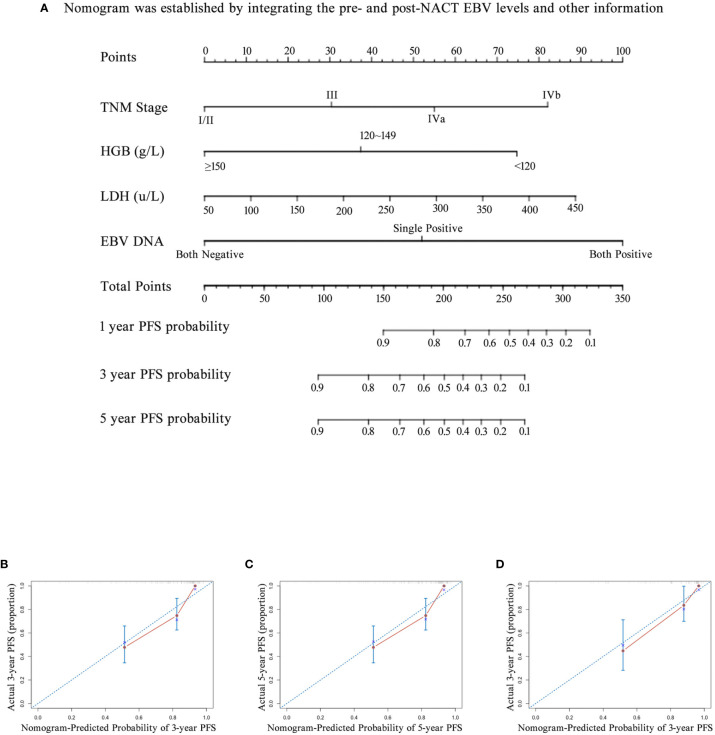
The nomogram and its calibration curve established by using pre- and post- neoadjuvant chemotherapy (NACT) EBV levels. **(A)** Nomogram; The calibration curves for predicting patient PFS at **(B)** 3-year and **(C)** 5-year in the primary cohort and **(D)** 3-year in the validation cohort. Actual PFS is plotted on the y-axis; nomogram-predicted probability of PFS is plotted on the x-axis. PFS, progression-free survival.

We also investigated whether the combination of pre- and post-NACT EBV DNA levels can improve the accuracy of the prognostic model. EBV DNA levels were classified as double-positive, single-positive, or double-negative. This classification method based on EBV DNA levels significantly improved the prognostic accuracy of the nomogram for PFS, providing C-indices of 0.791 (95% CI, 0.728–0.854: **Figure 6A**) in the primary cohort and 0.819 (95% CI, 0.735–0.903) in the validation cohort (**Table 5**). C-indices of EBV alone were also improved, which were 0.710 (95% CI, 0.622–0.798) in the primary cohort and 0.738 (95% CI, 0.624–0.852) in the validation cohort. The calibration curves confirmed the high prognostic power of the combination of pre-NACT and post-NACT EBV DNA levels (**Figures 6B–D**).

**Table 5:** The C-indices of nomograms, TNM stage + HGB + LDH, and combined pre- and post-NACT EBV DNA for the prediction of the progression-free survival (PFS) in the primary cohort and validation cohort.

Factor	Primary cohort		Validation cohort	
	C-index (CI)	*P*	C-index (CI)	*P*
nomogram	0.791 (0.728–0.854)	<0.001^***^	0.819 (0.735–0.903)	<0.001^***^
Stage+HGB+LDH	0.732 (0.650–0.814)	<0.001^***^	0.736 (0.624–0.848)	<0.001^***^
EBV	0.710 (0.622–0.798)	<0.001^***^	0.738 (0.624–0.852)	<0.001^***^

Nomogram A, including four high risk factors (Stage, HGB, LDH, and combined pre- and post-NACT EBV levels); C-index, concordance index; CI, confidence interval. N (Primary cohort) = 206; N (Validation cohort) = 76. ***P < 0.001; bold means statistically significant.

## Discussion

To the best of our knowledge, this is the first study combining pre-NACT and post-NACT plasma EBV DNA levels to predict patient prognosis. We found significant variations in the EBV DNA levels depending on the treatment stage. We also found that pre-NACT and post-NACT plasma EBV levels were a robust prognostic biomarker independent of the NACT regimen. Patients who were negative for EBV DNA before and after NACT had a better prognosis than EBV DNA-positive patients. Notably, EBV-positive to EBV-negative conversion after NACT was strongly associated with a favorable prognosis. The C-indices of the nomogram combining pre-NACT and post-NACT plasma EBV DNA levels were higher than those of other nomograms in the primary and validation cohorts.

Plasma EBV DNA levels are an accurate and reliable predictor of NPC progression. Changes in the plasma EBV DNA levels can provide insights into the relationship between EBV infection and NPC. Tang and Hong reported that pre-treatment EBV DNA levels were an important prognostic marker (6, 23). Similarly, Leung et al. demonstrated that plasma EBV DNA levels during radiotherapy predicted clinical outcomes (29). By monitoring the plasma EBV DNA levels at different stages of chemotherapy and radiotherapy, Rui et al. found that EBV DNA levels before, after, and during NACT predicted the risk of metastasis in patients with NPC (16). Consistently, we found that pre-NACT and post-NACT EBV DNA levels strongly predicted survival in patients with NPC. The 3-year PFS of patients with EBV DNA level decline during NACT was 31.8% higher than that of patients who remained EBV-positive after NACT. Detectable EBV DNA levels after first-line therapy were associated with local recurrence, distant metastasis, and disease progression, consistent with the findings of Lv et al. (25) These findings suggest that active EBV infection is associated with aggressive tumor phenotypes in NPC. EBV DNA decrease after NACT level might indicate early tumors response. As previously reported, NACT response is associated with a favorable prognosis in patients with NPC and can be used to risk-stratify patients (30, 31). Early tumor response is associated with size reduction in primary tumor lesions detected by imaging methods; however, biomarkers of early tumor response are lacking (32, 33). Our findings suggest that changes in EBV DNA levels during NACT may be a reliable biomarker of early response in patients with NACT. Consistently, Chen et al. used changes in EBV DNA levels and (18)F-FDG PET-derived parameters to evaluate the early response in patients with NPC (34).

Tang et al. found that the C-index of a nomogram with EBV DNA levels was significantly higher than that of a nomogram without an EBV load (23). Nomograms of EBV DNA levels can also predict tumor recurrence and survival in patients with NPC (35, 36). In another study, EBV DNA levels predicted metastasis within six months after treatment (37). Therefore, EBV DNA levels could be used to risk-stratify patients and guide clinical decision making (35). However, all previous studies used pre-treatment EBV DNA levels to predict the prognosis. However, our findings suggest that monitoring the changes in EBV DNA levels during treatment may be a better predictor of prognosis in patients with NPC. We found that the C-indices of the prediction model based on the EBV DNA levels both before and after NACT were higher than those of the traditional nomograms of EBV DNA levels only before or after treatment. Thus, the clinical implementation of combined pre-NACT and post-NACT EBV DNA testing may improve the prognostic accuracy in patients with NPC. Given the strong relationship between pre-NACT and post-NACT EBV DNA levels and treatment outcomes, we believe that dynamic plasma EBV DNA may serve as a valuable marker to help predict prognosis, as well as guide NPC screening and treatment (9, 38–40). According to our findings, NACT may be continued until EBV DNA levels have reached <400 copies/mL. In patients who persistently have high plasma levels of EBV DNA, aggressive treatments (e.g., EBV-targeted cytotoxic T lymphocytes) may be needed (41, 42).

The current study had a few limitations. Importantly, not all the patients had complete EBV test results for all treatment stages. Plasma EBV DNA levels at all four treatment stages were known for only 76 patients, and the small cohort size may have led to a sampling bias. Additionally, we did not take into account different ACT strategies. However, differences in ACT regimens may lead to different clinical outcomes. Additionally, the primary endpoint of the study, PFS, is unreliable in retrospective studies due to the expected inconsistency in determining the events other than death. Another limitation is that post-NACT, post-radiotherapy, and post-treatment EBV DNA levels were assessed between the last date of the former treatment and the first date of later therapy.

## Conclusion

We evaluated the relationship between the plasma EBV DNA levels and treatment outcomes in patients with NPC. Our findings suggest that the combination of pre-NACT and post-NACT plasma EBV DNA levels accurately predicts survival in patients with NPC. We also provided evidence that tracking plasma EBV DNA may benefit patients undergoing NACT for NPC. Future multicenter, randomized, controlled trials are required to confirm the prognostic value of EBV DNA levels in patients with NPC.

## Data Availability Statement

The original contributions presented in the study are included in the article/**Supplementary Materials**, further inquiries can be directed to the corresponding authors.

## Ethics Statement

The studies involving human participants were reviewed and approved by Regional Ethics Committee of Tongji Medical College, Huazhong University of Science and Technology. The patients/participants provided their written informed consent to participate in this study.

## Author Contributions

Conceptualization: ZZ and GP. Methodology: GP and LZ. Software: TO. Validation: YX, LB, and MQ. Formal analysis: LZ and QL. Investigation: LZ and TO. Data curation: QL. Writing—original draft preparation: LZ and TO. Writing—review and editing: GP and ZZ. Visualization: YX and LB. Supervision: QL, LB, and MQ. Funding acquisition: ZZ and GP. All authors contributed to the article and approved the submitted version.

## Funding

This work was supported by National Natural Science Foundation of China (Grant No. 81874232 and 82071067).

## Conflict of Interest

The authors declare that the research was conducted in the absence of any commercial or financial relationships that could be construed as a potential conflict of interest.

## Publisher’s Note

All claims expressed in this article are solely those of the authors and do not necessarily represent those of their affiliated organizations, or those of the publisher, the editors and the reviewers. Any product that may be evaluated in this article, or claim that may be made by its manufacturer, is not guaranteed or endorsed by the publisher.

## Supplementary Material

Supplementary Figure 1 Kaplan-Meier survival curves displaying progression-free survival (PFS) and overall survival (OS) of patients with different EBV status in different treatment stages. **(A)** Comparison of PFS and **(B)** OS between patients with EBV-DNA status changing from positive to negative and remaining positive after radiotherapy; **(C)** Comparison of PFS and **(D)** OS between patients with EBV-DNA status turning from negative to positive and remaining negative after radiotherapy; **(E)** Comparison of PFS and **(F)** OS between patients with EBV-DNA status switching from positive to negative and remaining positive after adjuvant chemotherapy (ACT); **(G)** Comparison of PFS and **(H)** OS between patients with EBV-DNA status turning from negative to positive and remaining negative after ACT.

Supplementary Figure 2 The relationship between neoadjuvant chemotherapy (NACT) regimens and prognosis in different subgroups. **(A)** PFS and **(B)** OS in group of pre-NACT; **(C)** PFS and **(D)** OS in group of post-NACT; **(E)** PFS and **(F)** OS in group of post-radiotherapy; **(G)** PFS and **(H)** OS in group of post-treatment. PFS, Progression-free survival (PFS); OS, Overall survival; DCF, docetaxel plus cisplatin and fluorouracil; GP, gemcitabine plus cisplatin; DP, docetaxel plus cisplatin.

Supplementary Figure 3 Nomogram for predicting patients’ 1-year, 3-year and 5-year progression-free survival (PFS). **(A)** The nomogram was established by integrating the TNM stage, HGB, LDH and post- radiotherapy EBV levels; **(B)** The nomogram was established by integrating the TNM stage, HGB, LDH and post-treatment EBV levels.

Supplementary Figure 4 The calibration curve of nomogram for predicting progression-free survival (PFS) by using post-radiotherapy EBV DNA levels or post-treatment EBV DNA levels. **(A)** 3-year and **(B)** 5-year in nomogram with post-radiotherapy EBV DNA levels in the primary cohort and **(C)** 3-year in the validation cohort; **(D)** 3-year and **(E)** 5-year in nomogram with post-treatment EBV DNA levels in the primary cohort and **(F)** 3-year in the validation cohort. Actual PFS is plotted on the y-axis; nomogram- predicted probability of PFS is plotted on the x-axis.

Supplementary Table 1 Patient demographic and clinical characteristics of inclusion patients, exclusion patients and validation patients.

Supplementary Table 2 NACT regimens of four subgroups in NPC patients with different EBV DNA levels. NACT, neoadjuvant chemotherapy; DCF, docetaxel plus cisplatin and fluorouracil; GP, gemcitabine plus cisplatin; DP, docetaxel plus cisplatin.
