# Supplementary figures and images for: Prognostic Value of Plasma Epstein-Barr Virus DNA Levels Pre- and Post-Neoadjuvant Chemotherapy in Patients With Nasopharyngeal Carcinoma
Source: Front Oncol. 2021 Sep 16;11:714433. doi: 10.3389/fonc.2021.714433 (PMC8543894; doi:10.3389/fonc.2021.714433)

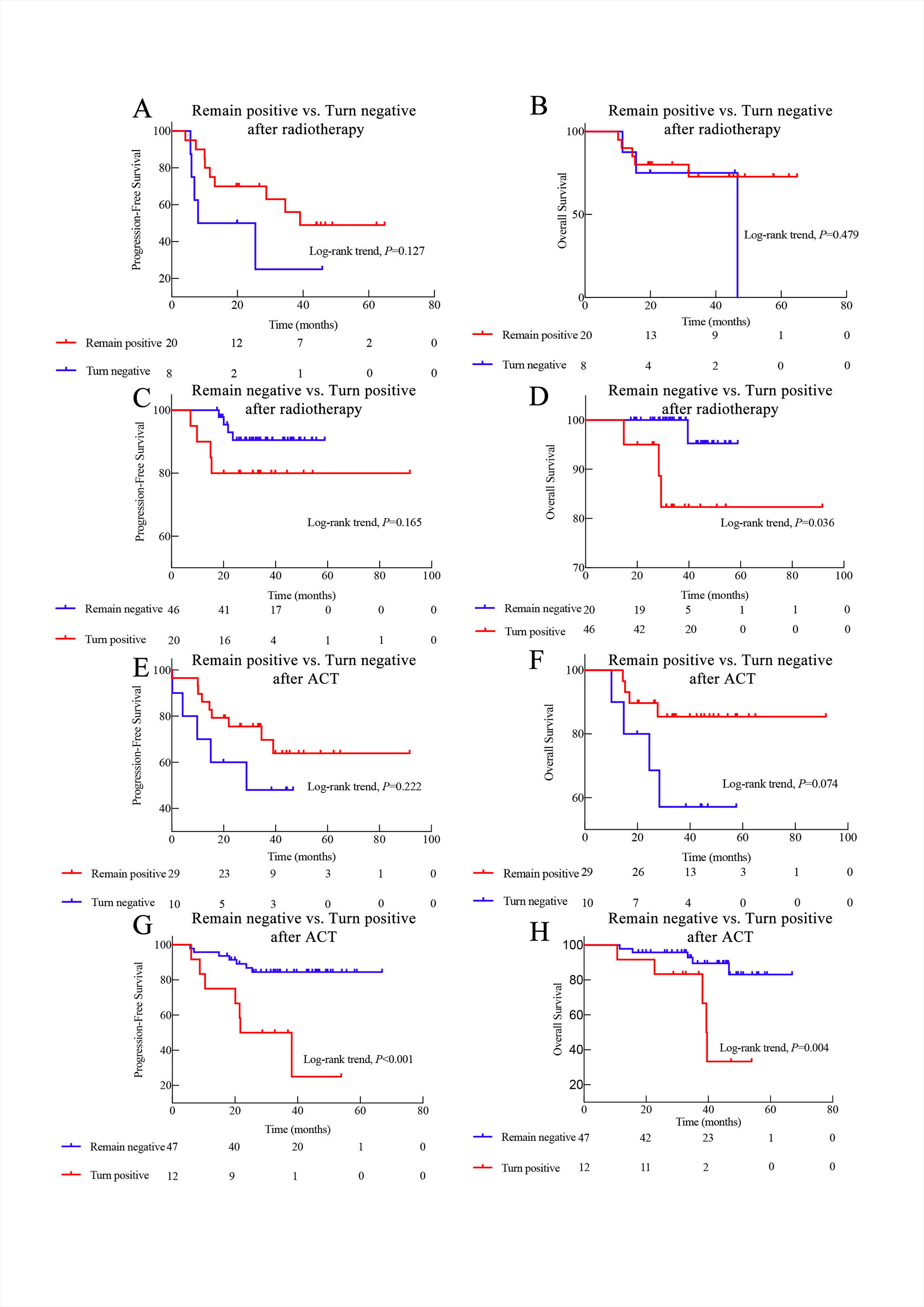

Supplement: Supplementary Figure 1 — Kaplan-Meier survival curves displaying progression-free survival (PFS) and overall survival (OS) of patients with different EBV status in different treatment stages. (A) Comparison of PFS and (B) OS between patients with EBV-DNA status changing from positive to negative and remaining positive after radiotherapy; (C) Comparison of PFS and (D) OS between patients with EBV-DNA status turning from negative to positive and remaining negative after radiotherapy; (E) Comparison of PFS and (F) OS between patients with EBV-DNA status switching from positive to negative and remaining positive after adjuvant chemotherapy (ACT); (G) Comparison of PFS and (H) OS between patients with EBV-DNA status turning from negative to positive and remaining negative after ACT. [file Image_1.tif]

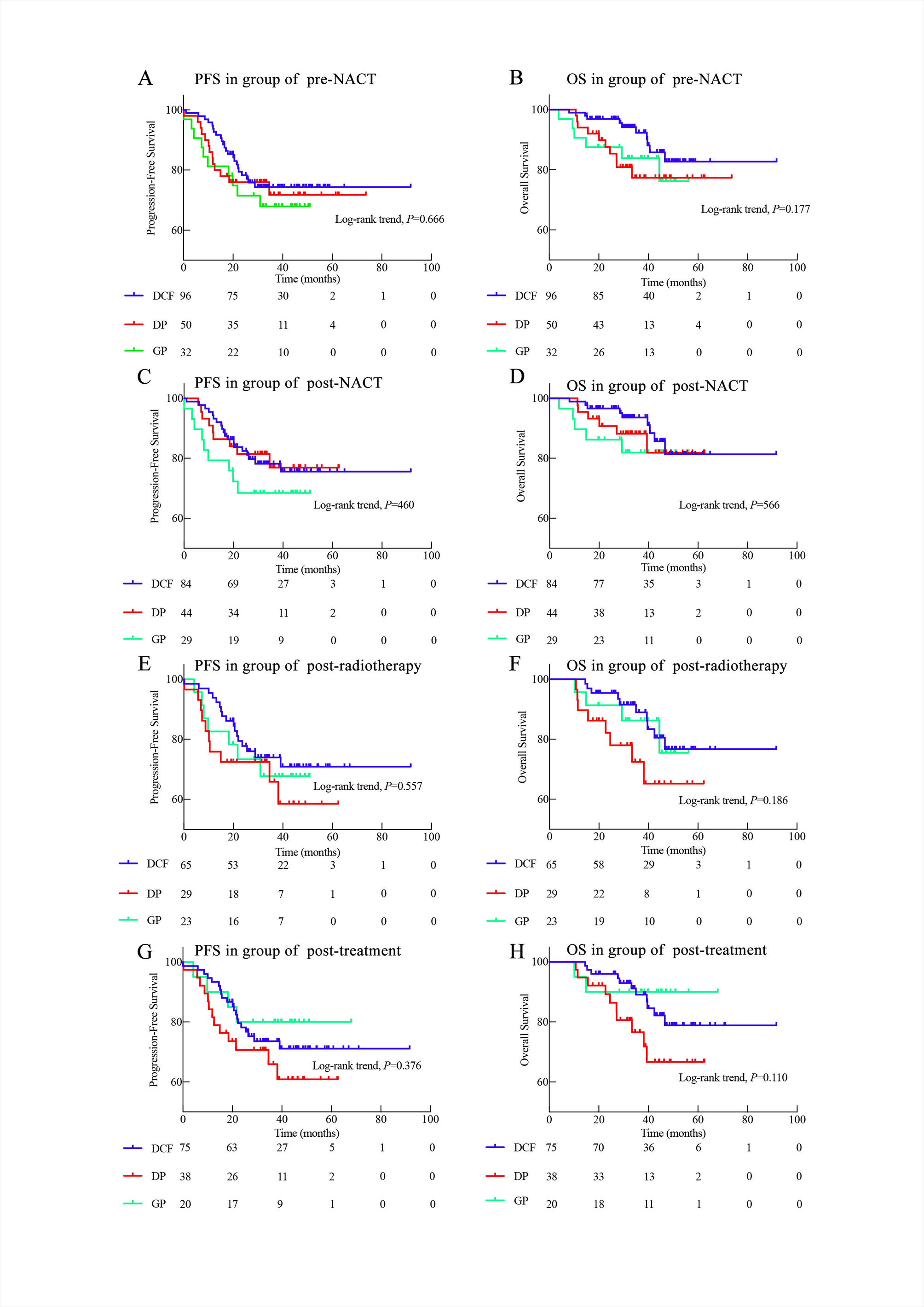

Supplement: Supplementary Figure 2 — The relationship between neoadjuvant chemotherapy (NACT) regimens and prognosis in different subgroups. (A) PFS and (B) OS in group of pre-NACT; (C) PFS and (D) OS in group of post-NACT; (E) PFS and (F) OS in group of post-radiotherapy; (G) PFS and (H) OS in group of post-treatment. PFS, Progression-free survival (PFS); OS, Overall survival; DCF, docetaxel plus cisplatin and fluorouracil; GP, gemcitabine plus cisplatin; DP, docetaxel plus cisplatin. [file Image_2.tif]

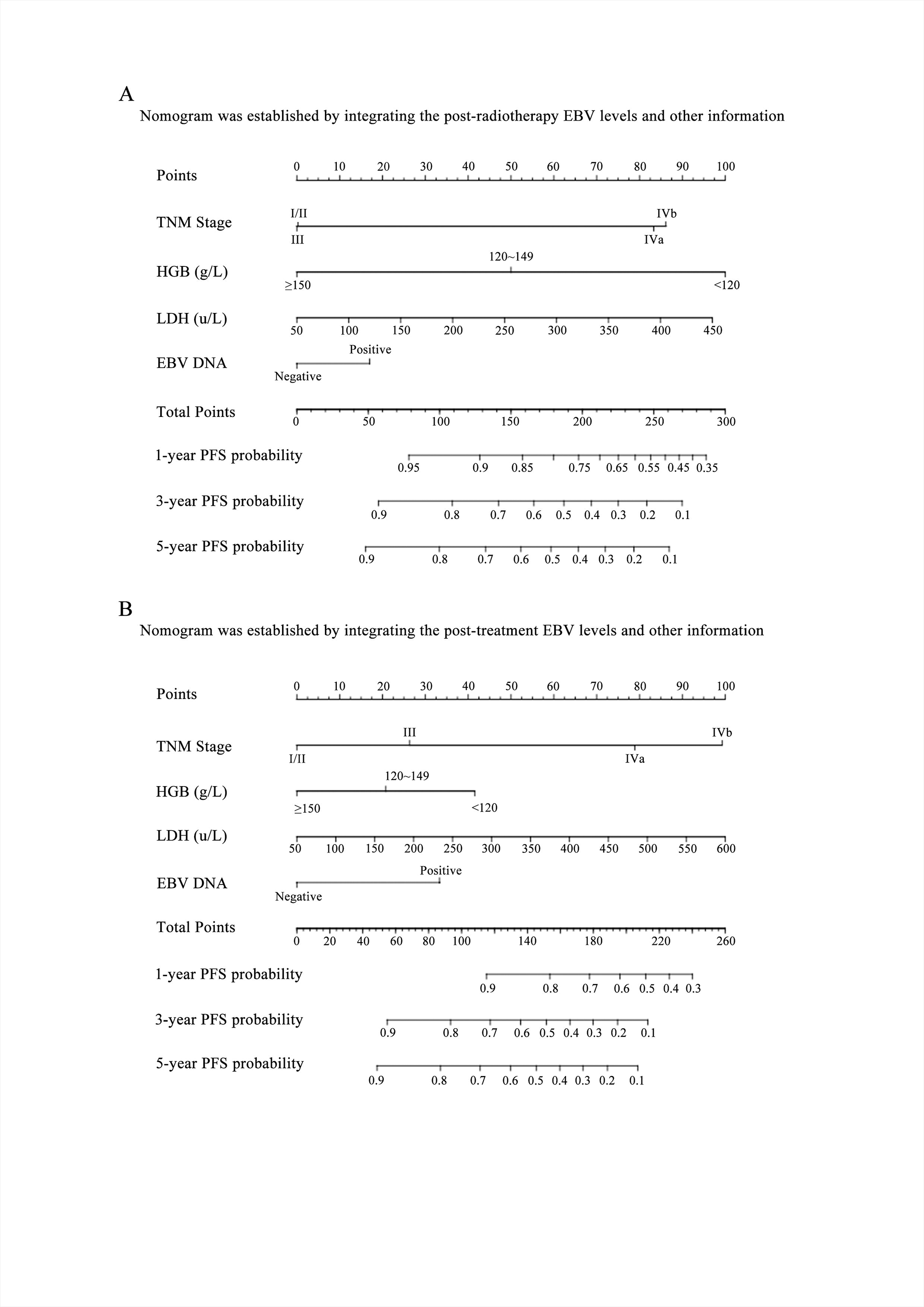

Supplement: Supplementary Figure 3 — Nomogram for predicting patients’ 1-year, 3-year and 5-year progression-free survival (PFS). (A) The nomogram was established by integrating the TNM stage, HGB, LDH and post- radiotherapy EBV levels; (B) The nomogram was established by integrating the TNM stage, HGB, LDH and post-treatment EBV levels. [file Image_3.tif]

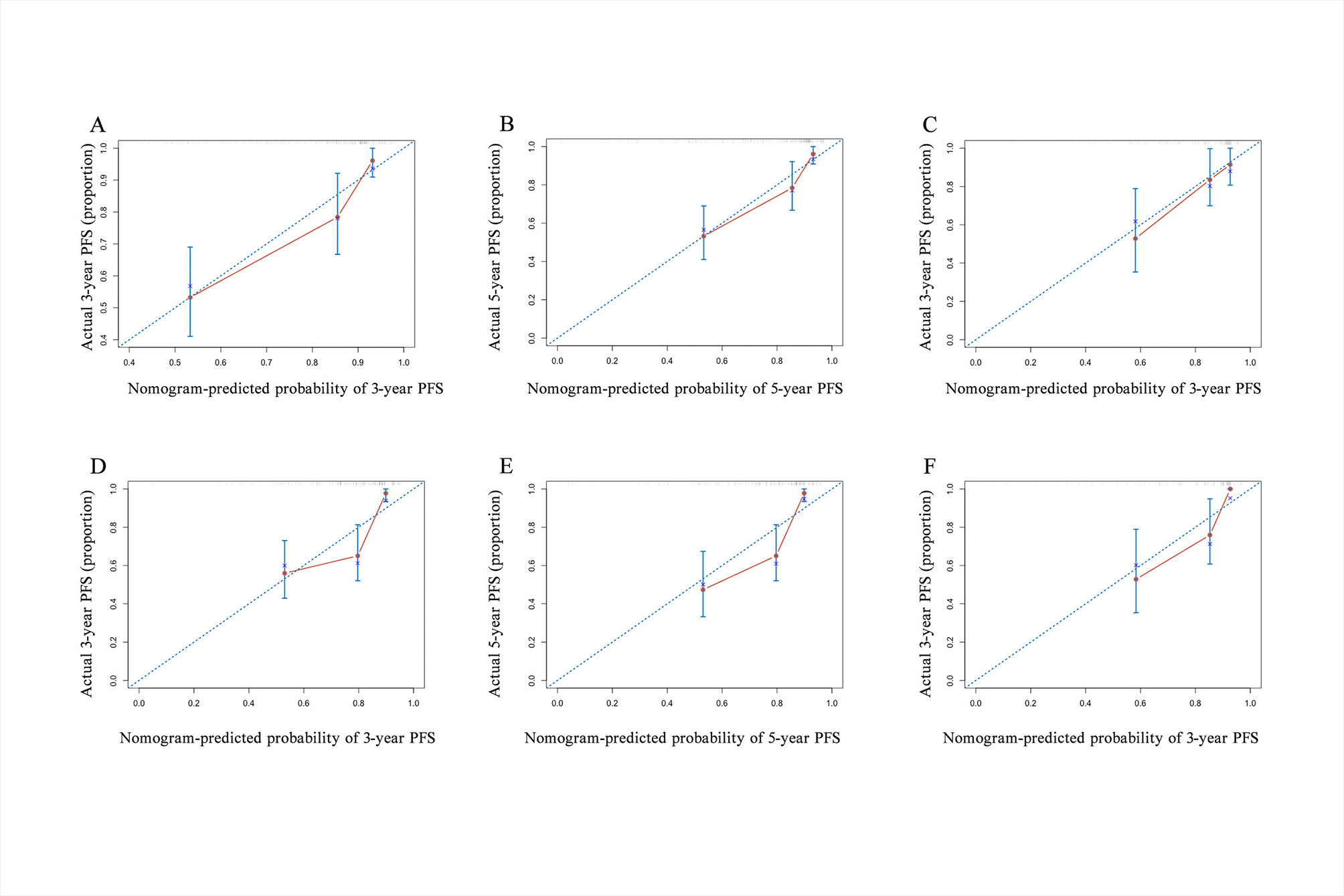

Supplement: Supplementary Figure 4 — The calibration curve of nomogram for predicting progression-free survival (PFS) by using post-radiotherapy EBV DNA levels or post-treatment EBV DNA levels. (A) 3-year and (B) 5-year in nomogram with post-radiotherapy EBV DNA levels in the primary cohort and (C) 3-year in the validation cohort; (D) 3-year and (E) 5-year in nomogram with post-treatment EBV DNA levels in the primary cohort and (F) 3-year in the validation cohort. Actual PFS is plotted on the y-axis; nomogram- predicted probability of PFS is plotted on the x-axis. [file Image_4.tif]
