# Supplementary material for: Prognostic Value of Plasma Epstein-Barr Virus DNA Levels Pre- and Post-Neoadjuvant Chemotherapy in Patients With Nasopharyngeal Carcinoma
Source: Front Oncol. 2021 Sep 16;11:714433. doi: 10.3389/fonc.2021.714433 (PMC8543894; doi:10.3389/fonc.2021.714433)
Supplement: Supplementary Table 1 — Patient demographic and clinical characteristics of inclusion patients, exclusion patients and validation patients. [file Table_1.docx]

Table S 1. Patient demographic and clinical characteristics of inclusion patients, exclusion patients and validation patients.

| Characteristic | Inclusion patients | Validation patients | Exclusion patients | *P* |
| --- | --- | --- | --- | --- |
|  | No. (%) | No. (%) | No. (%) |  |
| **Age, y** |  |  |  |  |
| ≤45 | 91 (44.2) | 30 (39.5) | 235 (48.0) |  |
| >45 | 115 (55.8) | 46 (60.5) | 255 (52.0) | 0.311 |
| **Gender** |  |  |  |  |
| Female | 49 (23.8) | 15 (19.7) | 108 (22.0) |  |
| Male | 157 (76.2) | 61 (80.3) | 382 (78.0) | 0.752 |
| **Clinical stage** |  |  |  |  |
| I/ II | 29 (14.1) | 11 (14.5) | 68 (13.9) | 0.989 |
| III | 87 (42.2) | 34 (44.7) | 220 (44.9) | 0.807 |
| IVa | 74 (35.9) | 22 (28.9) | 172 (35.1) | 0.527 |
| IVb | 16 (7.8) | 9 (11.8) | 35 (7.1) | 0.363 |
| **Tumor stage** |  |  |  |  |
| T1 | 3 (1.5) | 2 (2.6) | 6 (1.2) | 0.628 |
| T2 | 70 (34.0) | 24 (31.6) | 172 (35.1) | 0.823 |
| T3 | 78 (37.9) | 34 (44.7) | 200 (40.8) | 0.555 |
| T4 | 55 (26.7) | 16 (21.1) | 112 (22.9) | 0.470 |
| **Node stage** |  |  |  |  |
| N0 | 9 (4.4) | 4 (5.3) | 22 (4.5) | 0.942 |
| N1 | 57 (27.7) | 25 (32.9) | 147 (30.0) | 0.671 |
| N2 | 102 (49.5) | 34 (44.7) | 221 (45.1) | 0.545 |
| N3 | 38 (18.4) | 13 (17.1) | 100 (20.4) | 0.713 |
| **M stage** |  |  |  |  |
| M0 | 190 (92.2) | 67 (88.2) | 445 (90.8) |  |
| M1 | 16 (7.8) | 9 (11.8) | 45 (9.2) | 0.566 |
| **LDH, g/L** |  |  |  |  |
| <245 | 187 (90.8) | 70 (92.1) | 451 (92.0) |  |
| ≥245 | 19 (9.2) | 6 (7.9) | 39 (8.0) | 0.851 |
| **WBC, 10^9^/L** |  |  |  |  |
| <4 | 21 (10.2) | 7 (9.2) | 47 (9.6) | 0.959 |
| 4-10 | 173 (84.0) | 63 (82.9) | 406 (82.9) | 0.935 |
| ≥10 | 12 (5.8) | 6 (7.9) | 37 (7.6) | 0.695 |
| **HGB, g/L** |  |  |  |  |
| <120 | 33 (16.0) | 9 (11.8) | 69 (14.1) | 0.643 |
| 120-150 | 133 (64.6) | 53 (69。7) | 319 (65.1) | 0.670 |
| ≥150 | 40 (19.4) | 14 (18.4) | 102 (20.8) | 0.843 |
| **PLT, 10^9^/L** |  |  |  |  |
| <100 | 3 (1.5) | 0 (0.0) | 5 (1.0) | 0.562 |
| 100-300 | 174 (84.5) | 68 (89.5) | 426 (86.9) | 0.400 |
| ≥300 | 29 (14.1) | 8 (10.5) | 50 (12.0) | 0.659 |
| **Smoking** |  |  |  |  |
| No | 111 (53.9) | 45 (59.2) | 269 (54.9) |  |
| Yes | 95 (46.1) | 31 (40.8) | 221 (45.1) | 0.723 |
| **Alcohol** |  |  |  |  |
| No | 138 (67.0) | 50 (65.8) | 318 (64.9) |  |
| Yes | 68 (33.0) | 26 (34.2) | 172 (35.1) | 0.868 |
