# Supplementary material for: Prognostic Value of Plasma Epstein-Barr Virus DNA Levels Pre- and Post-Neoadjuvant Chemotherapy in Patients With Nasopharyngeal Carcinoma
Source: Front Oncol. 2021 Sep 16;11:714433. doi: 10.3389/fonc.2021.714433 (PMC8543894; doi:10.3389/fonc.2021.714433)
Supplement: Supplementary Table 2 — NACT regimens of four subgroups in NPC patients with different EBV DNA levels. NACT, neoadjuvant chemotherapy; DCF, docetaxel plus cisplatin and fluorouracil; GP, gemcitabine plus cisplatin; DP, docetaxel plus cisplatin. [file Table_2.docx]

Table S 2. NACT regimens of four subgroups in NPC patients with different EBV DNA levels.

| Factor | DCF | GP | DP | *P* |
| --- | --- | --- | --- | --- |
|  | No. (%) | No. (%) | No. (%) |  |
| **Pre-treatment** |  |  |  |  |
| Positive | 42 (43.8) | 13 (40.6) | 22 (44.0) |  |
| Negative | 54 (56.3) | 19 (59.4) | 28 (56.0) | 0.946 |
| **Post-NACT** |  |  |  |  |
| Positive | 24 (27.3) | 9 (31.0) | 16 (36.4) |  |
| Negative | 64 (72.7) | 20 (69.0) | 28 (63.6) | 0.562 |
| **Post-radiotherapy** |  |  |  |  |
| Positive | 28 (43.1) | 11 (47.8) | 9 (31.0) |  |
| Negative | 37 (56.9) | 12 (55.2) | 20 (69.0) | 0.417 |
| **Post-treatment** |  |  |  |  |
| Positive | 31 (41.3) | 7 (35.0) | 17 (44.7) |  |
| Negative | 44 (58.7) | 13 (65.0) | 21 (55.3) | 0.774 |
